# 

**Published:** 1868-12-15

**Authors:** 


### CHICAGO MEDICAL JOURNAL.

J. ADAMS ALLEN, M.D., LL.D., Editor. WALTER HAY, A.M., M.D., Associate Editor.

All letters for the JOURNAL should be addressed to Dr. J. ADAMS ALLEN, No. 71 Dearborn St., Chicago, Ill.

TERMS — $3 Per Annum, in Advance;

$4 if not paid before 1st of July, in each year. Single copies, 25 cents.

### INDEX FOR VOL. XXV.—1868.

Abstract of Medical Science, Half-yearly .......... 110
Abortion, a Cause of Insanity .......... 658
Acid, Acetic .......... 26
Acid, Thymic .......... 668
Advertising Specialties .......... 111, 182
Address (Prof. Blaney) .......... 115
American Surgery, Recognition of Abroad .......... 219
Anatomical Specimens, etc. .......... 82
Anatomical Specimens, Preserving of .......... 280
Annual Abstract of Therapeutics and Materia Medica .......... 110
Anus, Occluded .......... 270
Apoplexy .......... 628
Apologetic .......... 708
Arrearages .......... 511
Artery Innominatum, Ligation of .......... 74
Associations .......... 472
Association, American Pharmaceutical .......... 213
Atomizing Apparatus .......... 613

Back Numbers .......... 181
Bean Calabar, in Tetanus .......... 398
Bi-Sulphate of Lime in Beef Soup .......... 64
Biennial Retrospect of Medicine and Surgery .......... 21
Biliary Calculi .......... 497
Blood Corpuscles, Human, New Feature in .......... 315
Bliss & Sharp .......... 183
Bright's Disease .......... 24
Brown, Cod Liver Oil .......... 25
Braithwaite's Retrospect .......... 110
Brainard Medical Society .......... 314

Calabar Bean in Traumatic Tetanus .......... 398
Carson's Synopsis .......... 82
Cases in Practice .......... 176
Carcinoma of the Stomach .......... 177
Carcinoma of the Spleen .......... 729
Card .......... 272
Calcareous Degeneration of Pericardium .......... 321
Carbolic Acid and Creosote .......... 659
Carbolate of Quinia .......... 662
Cataract .......... 665
Carr, Prof. E. S. .......... 739
Chicago, Past, Present, and Future .......... 575
Circular .......... 181
Clinical Cases .......... 58, 165, 306
Clippings from Correspondence .......... 546
Clinical Lectures, Eye and Ear .......... 314, 614

Clinic, Obstetric .......... 23
Correspondence ..........58, 435, 465, 468, 505, 532, 567, 568, 571, 651
Correspondence, Foreign..........75, 262, 300, 323, 347, 461, 537, 542, 606
Correspondence, Philadelphia ....303, 327, 351, 464, 572, 612, 640, 705, 772
Correspondence, Cincinnati..........305, 396
Correspondents, to .......... 29
Commencement Rush Medical College .......... 81
Conferring of Degrees .......... 113
Consumption in New England and Elsewhere .......... 213
Constitutions, etc., Chicago College of Pharmacy.......... 214
Contributions Relating to Causation of Disease, etc .......... 408
College Fees (Medical) .......... 645
Consumption, Causes and Cure.......... 214

Delirium Tremens .......... 54
Decolorizing Tincture of Iodine .......... 278
Delay in Publication.......... 613
Diseases of Lungs, etc .......... 21
" Heart .......... 23
" Children, Practical Treatise on .......... 23
" Larynx, Chronic.......... 80
" Children, Condie.......... 81
" Women .......... 212
" " Practical Treatise on.......... 313
" Venereal, Atlas of .......... 313, 329
" Eye, Treatise on.......... 313
" Skin .......... 361
" Digestive Organs, Functionally Treated.......... 361
Dislocation of Metarsal Bones.......... 73
Diagnosis, Mistaken Case of.......... 406
Diagnosis, Physical Lessons in.......... 468
Diphtheria, Treatment of.......... 405
Duns.......... 183
Duties of Medical Profession.......... 313
Dysentery .......... 652
Dystochia from Rare Position of Fœtus .......... 753

Ear, Treatment of Polypus of.......... 180
Editorials.......... 28, 62, 81, 111, 181, 215, 271, 344, 409, 440, 472, 507, 544, 576, 668, 708, 739
Education of Heart .......... 212
Elixirs of Calisaya, Iron, etc.......... 625
Endoscope .......... 111
Epidemic, Cholera, etc., Report on .......... 469
Epilepsy .......... 666
Errata .......... 355
Erudition, Medical .......... 205
Extracts, Fluid .......... 272, 393, 655
Extra Uterine Fœtation .......... 417
Experimental Medicine.......... 251
Extra Eight Pages.......... 511
Experimental Physiology .......... 475, 515
Experiments on Calculi .......... 734

Fees, Medical College .......... 645
Femur, Fracture of Neck of .......... 567
Foreign Notes and Excerpta .......... 513, 579, 616
Free Medical Education.......... 645

Fracture of Vertebra .... 431

Good Fellow, Inconvenience of being .... 510
Gangrene, Senile .... 202
General Practice, Notes, etc. in .... 499, 529
Gentian Root as a Dilator .... 185
Gelseminum, Fluid Extract of, Poisoning by .... 760
Gettysburg Katalysine Water .... 709
Gonorrhœal Rheumatism .... 24
Gossip .... 472, 544
Good Man's Legacy .... 214

Half-yearly Compendium of Medical Science .... 110
Hæmorrhage, Accidental .... 268
Hegeman & Co .... 740
Hernia, Strangulated Inguinal .... 597
History Completed .... 599
Hospital Reports .... 23
Hospital, U. S. Marine .... 613
Homœopathy *vs.* Common Sense .... 567
Honorary Degrees .... 613
Huson, W. B., Letter from .... 61
Hypodermic Injection of Quinia .... 63
Hygienic Treatment of Patients .... 166, 187
Hysteria .... 23

International Medical Congress .... 14, 29, 65
Invalids, Hotel for .... 313
Impacted Fracture of Neck of Femur .... 567
Inhalations, Treatment by .... 80
Inflammation, Modern Treatment of .... 686
Iodine and Carbolic Acid .... 185
Issue, the Next .... 32
Items .... 80, 182

Jucunde .... 31

Klinik der Ohrenkrankheiten .... 575
Kidney, Minute Investigation of .... 660

Larynx, Chronic Diseases of .... 80
Laceration of Perineum .... 761
Leucocytes, Spontaneous Development of in Blastemata, Origin of, etc. 679
Legacy, the Good Man's .... 214
Legitimate and Illegitimate Medicine .... 457
Lessons in Physical Diagnosis .... 468
Letter from J. T. Newman .... 61
" W. B. Huson .... 61
" Samuel Cole .... 300, 537
" A. Catron .... 465
" P. Curran .... 468
" A. J. Miller .... 532
" B. F. Lightfoot .... 571
" S. J. Jones .... 606
" E. K. Hutchins .... 612
Locomotor Ataxia .... 575
Loot .... 26, 63, 82, 185, 216, 318, 276, 658, 741
Lungs, Diseases of .... 21

Materia Medica for Use of Students.............................. 408
Mammary Cancer.................................................. 25
Mechanical Therapeutics ........................................ 20
Medical Association, American.........................344, 280
" Societies, Champaign County............................. 314
" " Chicago..................207, 314, 332, 410, 688, 777
" College, Rush........................113, 362, 433, 670, 740
" Erudition.................................................... 205
" Imbroglio.................................................... 671
Medicine, Experimental......................................... 417
Medicine, Practice of by Statute............................... 112
Memorandum...................................................... 315
Metatarsal Bones, Dislocation of................................ 73
Michigan University — The Bribe Accepted........................ 272
Midwifery, Treatise on.......................................... 573
Milk for Babes.................................................. 276
Mistaken Diagnosis ............................................. 406
Mutilation, Self................................................ 429
Monstrosity..................................................... 568

Necrosis, After-Treatment in.................................... 17
Nerves, Influence of Section of................................. 444
News Items.............................271, 308, 472, 673, 775
Nutrition....................................................... 27

Obituary.............................................474, 741
Obstetrics, Clinic.............................................. 23
" American Journal of...................................... 409
" Researches in............................................. 361
Obstetrical Cases, Remarks on................................... 639
Occluded Anus................................................... 270
Oil, Cod Liver, Fougera's....................................... 542
Opium and Stramonium............................................ 758
Ophthalmiatrische Beobachtungen................................. 22
Ovariotomy...................................................... 62

Painless Miscarriage............................................ 473
Passage of Sanguineous Corpuscles through Walls of Vessels........ 647
Pathogenesis—Pathological Anatomy, etc., of Disseminated Chronic Pneumonia, etc................................... 583, 615
Peccavimus...................................................... 512
Pepsine in Vomiting from Pregnancy.............................. 27
Periodical Headache............................................. 315
Periodicals Announced........................................... 545
Physiology, Pathological........................................ 144
Philadelphia School of Examinations............................. 315
Plastics........................................................ 80
Placenta Previa, Remarkable Case of............................. 493
Potash, Permanganate of......................................... 26
Polypus of Ear, Nature and Treatment of......................... 180
Pregnancy, Signs, etc., of...................................... 79
Progressive Locomotor Ataxia.................................... 575
" Paralysis of Insane.......................283, 379, 411
Prophylaxis of Venereal Diseases................................ 451
Prescriptions of Dr. Habershon.................................. 474
Prolonged Retention of Life by Infant........................... 638
Prevention of Disease, etc...................................... 408
Publication, Delay of........................................... 613

Pulmonary Phthisis, Lectures on .......... 361

Quinine, etc., in Scarlatina.......... 63
" Hypodermic Injection of .......... 63
" Carbolate of .......... 662

Retrospect, Braithwaite's.......... 110
Retrospect of Medicine and Surgery, Biurnal .......... 21
Relations between Sense of Touch, etc.......... 57
Response, by Class.......... 119
Report to New Sydenham Society .......... 214
Reply to Case of Instrumental Delivery.......... 260
Receipts since January 1st, etc.......... 282
Retroversion of Pregnant Uterus .......... 447, 559
Report on Epidemic Cholera, etc.......... 469
Rea's (Prof.) Valedictory.......... 121
Responsibility .......... 646
Rheumatism, Gonorrhœal .......... 24
Rhus Toxicodendron.......... 24
Richmond Medical Journal .......... 312

Sanitary Institutions, etc.......... 332
Senile Gangrene .......... 202
Self Mutilation.......... 429
Shoulder Presentation.......... 258
Simulated Epilepsy .......... 443
Skin, Diseases of .......... 361
Spermatorrhœa .......... 23
Spring Course of Instruction.......... 111, 433
Spongiose .......... 302
Splint Suspensory .......... 198
Sulphites, Antiseptic Properties of.......... 658
Sulphuric Ether, Use of, for Tapeworm.......... 26
Surgical Notes, Selections from .......... 301
Syphilis.......... 33
Syphilization .......... 488

Tetanus, Traumatic, Successfully Treated.......... 398
Therapeutics and Materia Medica .......... 331
Thymic Acid.......... 668
Tuberculosis, Virulence, etc., of.......... 711
Typhoid Fever, Abortive Treatment of.......... 25
Twenty-fifth Volume.......... 28

Uterus, Present Raid on.......... 30
Uterine Displacements .......... 70
" Diseases .......... 640

Valedictory, Prof. Rea's.......... 121
Vaginismus .......... 664
Venereal Disease, Prophylaxis of.......... 451
Vertebra, Fracture of.......... 431
Vessicular Bronchitis .......... 663

Western Tripod of Medicine .......... 506

